# Genetic Variants in MiRNA Processing Genes and Pre-MiRNAs Are Associated with the Risk of Chronic Lymphocytic Leukemia

**DOI:** 10.1371/journal.pone.0118905

**Published:** 2015-03-20

**Authors:** Idoia Martin-Guerrero, Angela Gutierrez-Camino, Elixabet Lopez-Lopez, Nerea Bilbao-Aldaiturriaga, Maria Pombar-Gomez, Maite Ardanaz, Africa Garcia-Orad

**Affiliations:** 1 Department of Genetics, Physical Anthropology and Animal Physiology, Faculty of Medicine and Odontology, University of the Basque Country, UPV/EHU, Bilbao, Spain; 2 Hospital Txagorritxu, Vitoria, Spain; 3 BioCruces Health Research Institute, UPV/EHU, Leioa, Spain; East Carolina University, UNITED STATES

## Abstract

Genome wide association studies (GWAS) have identified several low-penetrance susceptibility alleles in chronic lymphocytic leukemia (CLL). Nevertheless, these studies scarcely study regions that are implicated in non-coding molecules such as microRNAs (miRNAs). Abnormalities in miRNAs, as altered expression patterns and mutations, have been described in CLL, suggesting their implication in the development of the disease. Genetic variations in miRNAs can affect levels of miRNA expression if present in pre-miRNAs and in miRNA biogenesis genes or alter miRNA function if present in both target mRNA and miRNA sequences. Therefore, the present study aimed to evaluate whether polymorphisms in pre-miRNAs, and/or miRNA processing genes contribute to predisposition for CLL. A total of 91 SNPs in 107 CLL patients and 350 cancer-free controls were successfully analyzed using TaqMan Open Array technology. We found nine statistically significant associations with CLL risk after FDR correction, seven in miRNA processing genes (rs3805500 and rs6877842 in *DROSHA*, rs1057035 in *DICER1*, rs17676986 in *SND1*, rs9611280 in *TNRC6B*, rs784567 in *TRBP* and rs11866002 in *CNOT1*) and two in pre-miRNAs (rs11614913 in miR196a2 and rs2114358 in miR1206). These findings suggest that polymorphisms in genes involved in miRNAs biogenesis pathway as well as in pre-miRNAs contribute to the risk of CLL. Large-scale studies are needed to validate the current findings.

## Introduction

Chronic lymphocytic leukemia (CLL) is the most frequent leukemia of adults in Western countries. It is characterized by the accumulation of monomorphic small round B lymphocytes in peripheral blood, bone marrow, spleen and/or lymph nodes [1]. CLL has been reported to have the highest genetic predisposition of all hematologic neoplasias [2], with an ~8.5-fold increased relative risk in first-degree relatives [3]. However, previous genetic linkage studies failed to provide evidence that rare, high-penetrance genes contribute significantly to the familial risk [4], suggesting that additional loci of modest effects could be involved. Actually, recent genome-wide association studies (GWAS) have identified several low-penetrance susceptibility alleles associated with CLL [5–9], associations that have been thoroughly validated in multiple independent series [10–13]. Nevertheless, these studies have focused their effort on identifying genes involved in the risk of CLL and scarcely study regions that are implicated in non-coding molecules, such as microRNAs (miRNAs).

MiRNAs are a group of small, functional, non-coding RNAs of approximately 20 nucleotides that regulate gene expression at the post-transcriptional level by binding to the 3´ untranslated region (UTR) of a target gene [14]. This can lead to inhibition of translation or enhanced degradation of their target mRNAs. Through this mechanism, miRNAs can regulate a large number of genes. Consequently, variations in miRNA function can affect many targets with functional consequences.

To date several reports have described altered expression of miRNAs in CLL, suggesting their implication in the development of the disease [15–17]. MiRNA expression levels can be altered by genetic polymorphisms in pre-miRNAs and/or also in miRNA biogenesis genes. Additionally, genetic polymorphisms in miRNAs or in mRNA targets altering the mRNA-miRNA binding may be also relevant, affecting the miRNA function. Indeed, mutations in miRNA transcripts have been seen to be common in CLL [17, 18], suggesting that CLL predisposition involves genetic alterations in miRNAs. Supporting this idea, a recent study has found that certain miR-SNPs show different frequency in CLL patients compared with a control population [19].

In addition, several studies have hitherto described polymorphisms in miRNAs associated with the susceptibility to different malignancies. For example, rs7372209 in miR26a-1 has been associated with the risk of throat and esophageal cancer and rs11614913 in pre-miR196a2 with breast, lung and gastric cancers, etc [14]. However, despite accumulating evidence that inherited genetic variation in miRNA genes and miRNA processing genes can contribute to the predisposition for cancer, the role of these in the risk of CLL has not been extensively studied. Therefore, the aim of this study was to evaluate whether polymorphisms in pre-miRNAs, and/or miRNA processing genes, contribute to the risk of CLL.

## Methods

### Study population

The study population included a total of 523 Spanish individuals, 132 CLL patients previously reported [20] and 391 cancer-free controls. Both, cases and controls, were selected from the same residing area of the north of Spain and had West European ancestry. A brief description of the clinical characteristics of CLL population is described in S1 Table.

### Ethics Statement

CLL samples were obtained from the C. 0001174 collection and control samples from C.0001171 collection, registered in the Institute of Health Carlos III. All samples were collected before 2007, when Biomedical Research Act 14/2007 (http://www.boe.es/boe/dias/2007/07/04/pdfs/A28826-28848.pdf) was released in Spain. Medicine and Odontology Faculty of UPV/EHU ethics committee board approval was obtained.

### Genes and polymorphisms selection

A total of 118 SNPs were selected for the study. Of them, 72 SNPs were located in 21 genes involved in the miRNAs biogenesis and processing (S2 Table) and 46 SNPs were situated in 44 miRNAs (S3 Table). The SNPs in miRNA processing genes were selected after literature review and by using Patrocles database (http://www.patrocles.org). In each gene, using tagSNPs, we covered almost all the SNPs with potentially functional effects using bioinformatics tools (F-SNP, Fast-SNP, polymirTS [21, 22] Patrocles [23], Hapmap, Haploview). We considered functional effects those causing amino acid changes, alternative splicing, those located in the promoter region in putative transcription factor binding sites, or disrupting/creating miRNAs targets. We also selected SNPs from the literature. Only SNPs with a reported minor allele frequency greater than 5% (MAF≥0.05) in European/Caucasoid populations were selected.

For the selection of SNPs in miRNAs, we had two factors in consideration. A) miRNAs can regulate a wide range of genes that are not completely defined. Therefore, any miRNA could be implicated in the regulation of genes affecting CLL risk. B) The number of miRNAs with polymorphisms described in the databases at the moment of selection with a MAF > 0.01 was affordable. Consequently, we selected all the SNPs in pre-miRNAs with a MAF>0.01 in European/Caucasoid populations, using Patrocles and Ensembl databases (http://www.ensembl.org/) (Welcome Trust Genome Campus, Cambridge, UK) and literature review. The preliminary list of SNPs was filtered using suitability for the Taqman OpenArray platform as criteria.

### Genotyping

Genomic DNA was extracted from peripheral blood from controls and from granulocytes (normal cells) from CLL patients as previously described [20]. SNP genotyping was performed at the General Research Services (SGIker) of the University of the Basque Country using TaqMan Open Array technology (Applied Biosystems), following the manufacturer’s protocol. Data were analyzed with Taqman Genotyper software for genotype clustering and calling. As a genotyping control, 20 duplicate samples were placed across the plates.

### Statistical analysis

Haploview 4.2 was used to search for any deviation of Hardy-Weinberg equilibrium in controls, determine haplotype block structure, calculate the genotyping success rate for each SNP and compare haplotype frequencies and clinical parameters between cases and controls. Significance of the best result was corrected for multiple testing by 1000 permutations. Genotype frequencies in the cases and controls were compared using a χ2 test. The effect sizes of the associations were estimated by the OR’s from univariate logistic regression. The most significant test among the different genetic models was used to determine the statistical significance of each SNP. The results were adjusted for multiple comparisons by the False Discovery Rate (FDR) [24]. In all cases the significance level was set at 5%. Analyses were performed by using R v2.11 software.

### miRNAs secondary structures prediction

The RNAfold web tool (http://rna.tbi.univie.ac.at) [25] was used to predict the most stable secondary structures of the miRNAs showing significant SNPs. The analyzed sequences included 50 bp upstream and 50 bp downstream of the pre-miRNAs.

## Results

### Genotyping results

Successful genotyping was obtained in 457 DNA samples (87.4%); 107 of 132 CLL patients (81.1%) and 350 of 391 controls (89.5%). Of 118 SNPs, 91 (77.12%) were genotyped satisfactorily, 57 located in miRNAs processing genes (62.6%) and 34 in pre-miRNAs (37.4%). 27 SNPs were removed from the association study due to genotyping failures, deviations from HWE or because they were not polymorphic in our population (S4 Table). The average of success genotyping rates for all 91 SNPs was 96.2%.

### Genotype association study

**Fig 1:**
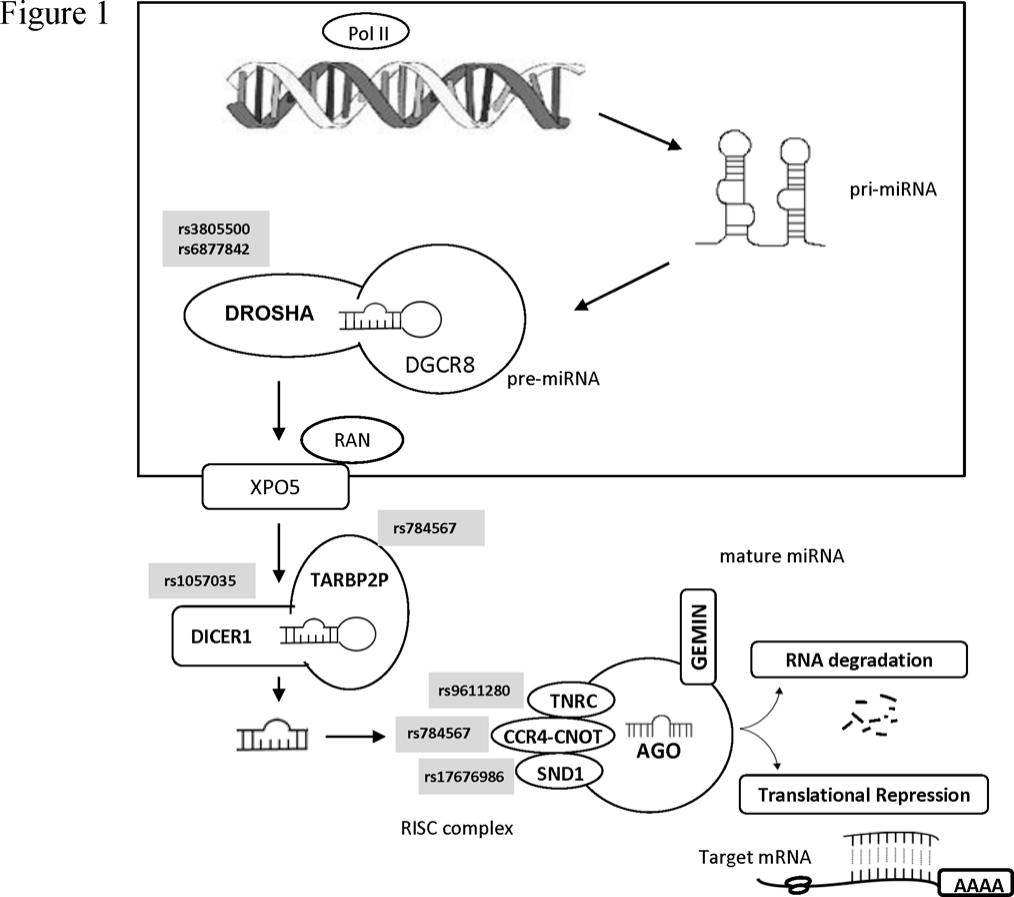
Overview of the significant SNPs in the miRNA network.

A total of 28 SNPs showed statistically significant association with CLL risk (S5 Table). After FDR correction, 9 SNPs remained significant. Among them, seven were located in six processing genes (Table 1, Fig. 1) and two in pre-miRNAs (Table 2). Two of the significant SNPs, rs3805500 and rs6877842, were located in *DROSHA* gene. Rs3805500 showed the most significant finding of the analyses under the log-additive genetic model (AA *vs* AG *vs* GG). The G allele showed a 0.54-fold decreased risk of CLL (95% CI: 0.38–0.77; *P* = 0.032). Other 5 SNPs in processing genes, rs1057035 in *DICER1*, rs17676986 in *SND1*, rs9611280 in *TNRC6B*, rs784567 in *TRBP* and rs11866002 in *CNOT1*, showed statistically significant results (*P*<0.05).

**Table 1:** Association of significant miRNA Processing Gene SNPs and Chronic Lymphocytic Leukemia risk.

Gene	SNP	Best fitting Model	Genotype	Controls n (%)	Cases n (%)	OR (95% CI)	*P* value	Adjusted *P* value*
***DROSHA***	**rs3805500**	Additive	AA	125 (37.0)	55 (55.0)	Reference		
			AG	158 (46.7)	38 (38.0)	0.54 (0.38–0.77)	0.0004	0.032
			GG	55 (16.3)	7 (7.0)			
	**rs6877842**	Recessive	GG + CG	330 (96.8)	90 (88.2)	Reference		
				(231+99)	(64+26)		0.002	0.038
			CC	11 (3.2)	12 (11.8)	4.00 (1.71–9.37)		
***DICER1***	**rs1057035**	Recessive	TT + CT	308 (89.0)	78 (75.0)	Reference		
				(156+152)	(46+32)		0.0007	0.032
			CC	38 (11.0)	26 (25.0)	2.7 (1.55–4.72)		
***SND1***	**rs17676986**	Recessive	CC + CT	341 (98.8)	97 (92.4)	Reference		
				(246+95)	(69+28)		0.001	0.038
			TT	4 (1.2)	8 (7.6)	7.03 (2.07–23.84)		
***TNRC6B***	**rs9611280**	Recessive	GG + AG	343 (98.6)	97 (92.4)	Reference		
				(273+70)	(80+17)		0.003	0.043
			AA	5 (1.4)	8 (7.6)	5.66 (1.81–17.69)		
***TRBP***	**rs784567**	Dominant	AA	80 (23.7)	38 (39.2)	Reference		
			AG + GG	258 (76.3)	59 (60.8)	0.48 (0.30–0.78)	0.003	0.043
				(181+77)	(40+19)			
***CNOT1***	**rs11866002**	Codominant	CC	134 (38.7)	54 (52.4)	Reference		
			CT	174 (50.3)	33 (32.0)	0.47 (0.29–0.77)	0.004	0.049
			TT	38 (11.0)	16 (15.5)	1.04 (0.54–2.03)		

Abbreviations: OR, odds ratio; CI, confidence interval; S, significative; NS, no significative

* Adjusted for multiple comparisons using the False Discovery Rate (FDR) at 5% level

**Table 2:** Association of significant miRNA-related SNPs and Chronic Lymphocytic Leukemia risk.

miRNA	SNP	SNP position	Best fitting Model	Genotype	Controls n (%)	Cases n (%)	OR (95% CI)	*P* value	Adjusted *P* value*
miR196a2	**rs11614913**	Premature	Recessive	CC + CT	296 (85.8)	75 (72.1)	Reference		
					(137+159)	(35+40)		0.002	0.038
				TT	49 (14.2)	29 (27.9)	2.34 (1.38–3.95)		
miR1206	**rs2114358**	Premature	Dominant	AA	110 (31.6)	48 (47.1)	Reference		
				AG + GG	238 (68.4)	54 (52.9)	0.52 (0.33–0.82)	0.005	0.049
					(181+57)	(36+18)			

Abbreviations: OR, odds ratio; CI, confidence interval; S, significative; NS, no significative

* Adjusted for multiple comparisons using the False Discovery Rate (FDR) at 5% level

We also observed that two SNPs in pre-miRNAs were significantly associated with CLL risk, rs11614913 and rs2114358 in the miR196a2 and miR1206, respectively. The genotype TT rs11614913 was associated with a 2.3-fold increased risk under the recessive model (95% CI: 1.38–3.95; *P* = 0.002) while the genotypes AA and AG rs2114358 showed 0.52-fold protective risk to CLL under the dominant model (95% CI: 0.33–0.82; *P* = 0.005).

When the correlation between SNP alleles and clinical parameters were analyzed (Rai stage, morphology, levels of serum markers such as β2-microglobulin or lactate dehydrogenase, presence of genomic aberrations, marrow infiltration, presence of adenopathy, lymphocyte doubling time, residual minimum disease, and treatment) no significant association was found in CLL patients (S6 Table).

### Haplotype Association study

We conducted haplotype analyses for the miRNA-related genes with at least two statistically significant SNPs in this study before FDR correction, including *DROSHA*, *DICER1*, *DGCR8* and *TNRC6B*. Two haplotype blocks, one in *DROSHA* and another one in *DGCR8*, showed statistically significant differences in their frequency in cases *versus* controls (χ2 test; adjusted *P*-value<0.05 after permutation testing) (Table 3). The significant haplotype of *DROSHA* gene, formed by rs3805500-rs7735863-rs6884823 (AGG), was associated with increased risk (OR = 1.63; 95% CI: 1.17–2.27), while the haplotype of *DGCR8* composed by rs9606248-rs1640299 (AT) was found to confer a significant reduced risk (OR = 0.67; 95% CI: 0.49–0.91) of developing CLL.

**Table 3:** Significant Haplotypes in miRNA processing genes and Chronic Lymphocytic Leukemia Risk.

Gene	SNPs	Haplotype	Freq. Controls	Freq. Cases	Chi-Square	Adjusted *P*-value^a^	OR^b^ (CI 95%)
*DROSHA*	rs3805500-rs7735863-rs6884823	**AGG**	0.60	0.71	9.23	0.004	1.63 (1.17–2.27)
*DGCR8*	rs9606248-rs1640299	**AT**	0.49	0.39	7.0	0.03	0.67 (0.49–0.91)

^a^Adjusted *P*-value after permutation test

^b^OR values are referred to the rest of haplotype

### miRNAs secondary structures

**Fig 2:**
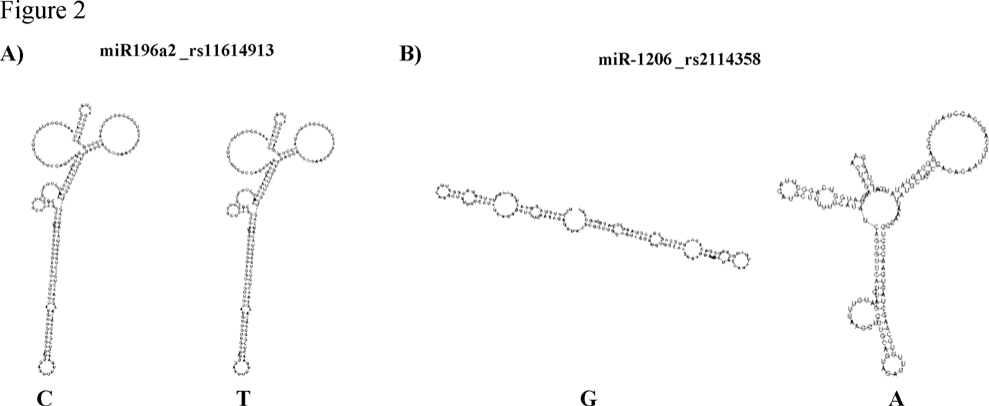
Secondary structures of the miRNAs showing significant SNPs. (A) miR196a2 and (B) miR1206, predicted by the RNAfold tool.

In order to check if SNPs in miR196a2 and miR1206 affected their secondary structure, we compared the minimum free energy for optimal secondary structures of both variants for each miRNA. The SNP rs2114358 in pre-miR1206 had a dramatic effect on its secondary structure, whereas the SNP rs11614913 in pre-miR196a2 had none (Fig. 2).

## Discussion

In the current study, we identified genetic variants in miRNA processing genes and pre-miRNAs that were associated with the risk of CLL. We examined a total of 91 SNPs, finding nine significantly associated after FDR correction, seven in miRNA processing genes and two in pre-miRNAs.

Interestingly, among the significant SNPs in miRNA processing genes, two were located in *DROSHA*; rs3805500 in the intronic region and rs6877842 upstream. Moreover, a haplotype including the SNPs rs3805500-rs7735863-rs6884823 (AGG) was also found significant in this gene. *DROSHA* encodes a type III Rnase enzyme, essential for the maturation of pri-miRNAs into pre-miRNAs [26]. Aberrant expression of *DROSHA* at mRNA or protein level was shown to be associated with patient survival and tumor progression in several types of cancer, including pleomorphic adenomas [27], esophageal cancer [28], cervical cancer [29] and ovarian cancer [30]. Moreover, the SNP rs3805500 which was the most significant finding in our study, was found by other authors to be associated with head and neck cancer recurrence [31]. In addition, a recent study in lung cancer has demonstrated that the polymorphism rs640831 in high LD with rs3805500 (r^2^>0.8) was associated with reduced *DROSHA* mRNA expression and with expression changes in 56 miRNAs out of 199 analyzed [32], some of which could also regulate genes involved in the development of CLL. Therefore, in the same way, the two polymorphisms detected in the present study could alter the expression of miRNAs involved in the regulation of genes involved in the risk of CLL.

Additionally, five extra miRNA biogenesis pathway SNPs were also found significant. One of them, rs1057035, was located within the 3’ UTR of *DICER1*, an important region for RNA stability. Moreover, it was located at the putative miRNA binding site of miR574-3p, which could affect the binding of this miRNA to *DICER*. DICER plays an important role in the cleavage of pre-miRNAs into their mature form. A recent study showed that rs1057035 C allele was associated with a lower expression of *DICER* and a decreased risk of oral cancer, which seemed to be due to a higher inhibition of miR574-3p on *DICER* mRNA [33]. In our study, in contrast, the variant genotype CC was associated with a 2.7-fold increased risk of CLL. These differences between studies suggest that *DICER* may have different roles in the tumorigenesis process depending on the cancer type. Supporting this idea, some studies have associated reduced expression of *DICER* with the development of lung cancer [34], while others have seen overexpression of *DICER* in oral cancer [35]. Therefore, it seems plausible that rs1057035 is affecting the binding of miR574-3p which in turn could be altering the regulation of *DICER* and the risk of CLL.

Another significant SNP, rs784567, was located in the 5' near gene region of *TRBP*, an integral component of a DICER-containing complex important to the cytoplasmic processing of pre-miRNAs into mature miRNAs. Previous studies also found rs784567 to be borderline significant in bladder cancer [36] and oral premalignant lesions [37]. Moreover, depletion of *TRBP* was found to result in significantly impaired miRNA biogenesis [38]. Consequently, genetic variation affecting the expression of this gene could lead to global changes in the miRNAome and subsequent deregulation of genes involved in CLL origin.

On the other hand, rs9611280 was situated in *TNRC6B*, gene that mediates miRNA-guided mRNA cleavage [39]. According to *in silico* analyses (F-SNP), this missense SNP was not predicted to abort protein function, but instead affected the regulation at splicing level. This change could perturb the maturation of the mRNA, changing the sequence of the protein, and therefore contribute to carcinogenesis in CLL.

Finally, the last two significant SNPs identified were rs17676986 at *SND1* and rs1186002 at *CNOT1*, genes involved in RNA-induced silencing complex (RISC). Genetic variants in both genes have been previously associated with autism [40] and ventricular arrhythmias [41] respectively, but this is the first time that are associated with cancer.

In addition to SNPs from miRNA processing genes, there were also two SNPs in miR196a2 and miR1206 significantly associated with CLL risk after FDR correction. miR196a2 rs11614913 variant homozygote TT showed an increased risk of CLL. Different rs11614913 genotype variants were previously reported to be associated with cancer risk; for example, the variant CC was associated with lung [42], hepatocellular [43], glioma [44], and gastric [45] cancer, while the variants TT or TT + CT were associated with breast [46], prostate [47], and head and neck cancer [48]. Despite these differences among studies, probably due to the different populations used in the analyses [49], it seems probable that rs11614913 plays an important role in cancer development. rs11614913 is located in the 3p mature miRNA region, so it could affect maturation of 5p and 3p miRNAs, as well as the expression and function of these miRNAs. Actually, Hoffman et al already demonstrated that rs11614913 C allele led to less efficient processing of the miRNA precursor to its mature form as well as diminished capacity to regulate target genes in breast cancer [46]. Interestingly, potential targets of miR196a2 include genes with relevance in CLL, such as *CDKN1B* or *HMOX1*. Therefore, alterations in the sequence of miR196a2 which may affect miRNA function, could contribute to CLL risk due to the deregulation of its targets.

Finally, miR1206 rs2114358 AG-GG genotypes showed a reduced risk of CLL. Remarkably, miR1206 is located at 8q24.2 a frequent site of chromosomal breaks in cancer and a signature of genomic instability [50]. Moreover, miR1206 is of particular interest as it is part of long non-coding RNA (lncRNA) transcript of the *PVT1* gene, locus of both tumorigenic translocations and retroviral insertions [51], which has been also shown to play a role in cell proliferation and apoptosis [52]. Overexpression of *PVT1* transcript has been documented in a variety of tumor tissues [53] and miR1206 expression has been found in increased levels in B cell tumors [54]. The allelic effect of miR1206 rs2114358 has been further analyzed in different human cell lines but without concluding results, suggesting that the effect of this SNP was cell line and cell type specific [55]. We found that miR1206 rs2114358 AG-GG genotypes showed a reduced risk of CLL, so it is likely to expect that AA genotype was associated with an increased risk. Moreover, we found by *in silico* analysis (RNA fold), that A allele altered the miRNA secondary structure, suggesting that this variant alter the maturation of miR1206.

A recent study found that rs56103835 in pre-miR323b was associated with CLL when compared with European samples from the 1000 genome project [19]. We also analyzed this SNP in our population, finding no significant association (S5 Table), neither when CLL population was compared with the European samples from the 1000 genome project (0.143 *versus* 0.187), nor when our control population was used (0.143 *versus* 0.183).

In conclusion, the findings of the present study indicated that SNPs located at *DROSHA*, *DICER1*, *SND1*, *TNRC6B*, *TRBP* and *CNOT1* genes, involved in miRNAs biogenesis pathway, as well as in miR196a2 and miR1206 may contribute to the risk of CLL. To our knowledge, this is the first study analyzing specifically miRNA-related SNPs in CLL, which opens a promising approach to search for new susceptibility markers in CLL. New large-scale studies including functional analyses will help to validate our findings.

## Supporting Information

S1 Table Clinical Data of the CLL Population. (XLS)

S2 Table Characteristics of the Single Nucleotide Polymorphisms in microRNA processing genes and selection criteria. (XLS)

S3 Table Characteristics of the Single Nucleotide Polymorphisms in microRNAs. (XLS)

S4 Table SNPs excluded from the association study. (XLS)

S5 Table Association of studied SNPs and Chronic Lymphocytic Leukemia risk. (XLS)

S6 Table Correlation between SNP alleles and clinical parameters. (XLS)
